# Meta-analysis comparing laparoscopic versus open resection for gastric gastrointestinal stromal tumors larger than 5 cm

**DOI:** 10.1186/s12885-017-3741-3

**Published:** 2017-11-13

**Authors:** Xiao Lian, Fan Feng, Man Guo, Lei Cai, Zhen Liu, Shushang Liu, Shuao Xiao, Gaozan Zheng, Guanghui Xu, Hongwei Zhang

**Affiliations:** Department of Digestive Surgery, Xijing Hospital, Fourth Military Medical University, 127 West Changle Road, 710032, Xi’an, Shaanxi China

**Keywords:** Gastrointestinal stromal tumor, Open resection, Laparoscopic resection, Meta-analysis

## Abstract

**Background:**

Data on the safety and feasibility of laparoscopic versus open resection for gastric gastrointestinal stromal tumors (GISTs) larger than 5 cm are limited. Therefore, the aim of this meta-analysis was to compared laparoscopic and open resection for gastric GISTs larger than 5 cm.

**Methods:**

We perform a literature search on PubMed, the Cochrane Library, and Embase. Review Manage version 5.1 (RevMan 5.1) was used for data analysis. The GRADE profiler software (version 3.6) was used to estimate the level of evidence.

**Results:**

A total of 6 observational studies and one unpublished retrospective cohort study met the inclusion criteria for the meta-analysis: 203 patients in LAP and 214 patients in OPEN group. The pooled result revealed that laparoscopic resection was associated with a same operative time (WMD = −0.87 min; 95% CI: -47.50 to 47.75; *P* = 0.97), intraoperative blood loss (WMD = −34.38 ml; 95% CI: -79.60 to 10.84; *P* = 0.14), overall complications (RR = 0.65; 95% CI: 0.38 to 1.12; *P* = 0.12), better 5-year disease-free survival (HR = 0.40; 95% CI: 0.17 to 0.91; *P* = 0.03) and overall survival (HR = 0.09; 95% CI: 0.02 to 0.40; *P* = 0.002) compared with open resection.

**Conclusion:**

Laparoscopic resection is a technically and oncologically safe and feasible approach for large-sized gastric GISTs (≥ 5 cm) compared to open resection.

**Electronic supplementary material:**

The online version of this article (10.1186/s12885-017-3741-3) contains supplementary material, which is available to authorized users.

## Background

Gastrointestinal stromal tumors (GISTs) are the most common mesenchymal tumors of the alimentary tract [1], and these tumors are generally characterized by high KIT expression [2]. GISTs originate from the interstitial cells of Cajal (ICC) because the immunophenotype of GIST cells is similar to that of ICCs [3]. GISTs can occur at any site throughout the alimentary tract but primarily occur in the stomach (60%–70%) [4]. The malignant potential of GISTs is associated with tumor size, tumor cell mitosis and differentiation [5].

Complete tumor excision with negative resection margins, avoiding tumor rupture and without lymphadenectomy, is the standard treatment for primary GISTs [6]. Simple wedge resection is also an adequate treatment for gastric GISTs when feasible. The development of minimally invasive surgery made gastric GISTs particularly amenable to laparoscopic resection [7]. A growing number of small size laparoscopic series [8–10] and several large scale meta-analyses [11–13] investigated the feasibility and safety of a laparoscopic versus open approach for small tumors. The National Comprehensive Cancer Network (NCCN) guidelines [14] recommend a laparoscopic approach for select GISTs located in favorable anatomical locations (e.g., greater curve or anterior wall of the stomach) by surgeons with appropriate laparoscopic experience. However, the European Society for Medical Oncology (ESMO) guidelines [6] clearly dissuade surgeons from preforming laparoscopic resection in patients with large-scale tumors because of the high risk of tumor rupture, which likely promotes relapse. Clinical practice guidelines for GISTs in Japan [15] suggest that the safe upper size of laparoscopic resection for gastric GISTs is less than 5 cm. Therefore, the safety and feasibility of laparoscopic resection for gastric GISTs larger than 5 cm remains ambiguous.

We initiated a comprehensive systematic review using meta-analysis to evaluate the current status of laparoscopic resection for gastric GISTs larger than 5 cm.

## Methods

A literature search was performed in December 2016. The primary searched sources were the PubMed, the Cochrane Library, and Embase. The following MeSH terms and their combinations were searched in [title]: gastric, GISTS/GIST/gastrointestinal stromal tumor/gastrointestinal stromal tumors, open/laparoscopic/laparoscopy and resection/surgery (Additional file 1). The links of every search result and all references in the original articles identified were reviewed to identify the additional literature that was not indexed. Two authors (XL and MG) independently screened potentially eligible studies. The following inclusion criteria were used: (1) primary article published in English and peer-reviewed journals; (2) the trial design compared laparoscopic and open resection for GISTs; (3) the available pathological and oncological data were listed separately for laparoscopic and open resection groups; and (4) the tumor size of the gastric GISTs included in analyses was larger than 5 cm. Two observers (LC and ZL) extracted the data using a unified datasheet, and a third observer (FF) was consulted when controversial issues arose. Extracted data included the following items: basic information of the study, clinicopathological features of objects, and perioperative and postoperative outcomes.

In addition to the published articles above, the screened unpublished retrospective data of gastric GISTs patients who received R0 resection in our center was involved in the meta-analysis. In order to improve the comparability of the data, we matched the 81 patients who underwent open resection to the 13 patients who received laparoscopic resection with a 1:1matched ratio. The matching condition was set to the tumor size difference between the two resection groups was no more than one centimeter (±1 cm). The detailed information about the exclusion criteria, surgical procedure, matched method, clinicopathological data and treatment plan was listed in Additional file 2.

The Methodological Index for Non-Randomized Studies (MINORS) was used to evaluate the methodological quality of the enrolled studies [16, 17]. The guideline consists of 12 items (Additional file 3) with a scoring system for each item of 0~2: 0 represented that the item was not reported in the article, 1 represented that the item was reported but deficiently; and 2 represented that the item was reported completely and appropriately. The total points available were 24 points. Point totals greater than 16 indicated high quality evidence, and scores lower than 16 indicated poor quality.

The GRADE system was used to evaluate the factors that influenced the quality and strength of recommendation of the evidence to rate the evidence quality for the four grades [18]: (a) high: further research is impossible to change our confidence in the estimate of the effect; (b) moderate: further research is possible to affect the reliability of the estimate of effect and may alter the assessment results; (c) low: further research is extremely likely to influence the confidence in the estimate of effect, and it is highly possible to change the assessment; (d) very low: we have little confidence in the estimate of the effect. Recommended levels were classified into “strong recommendation” and “weak recommendation”: a strong recommendation (or 1) indicated that the evaluators believed the intervention produced more benefit than harm; a weak recommendation (or 2) indicated that the pros and cons were not certain or equal regardless of the quality level of the evidence.

### Statistical analysis

The meta-analysis was performed according to the standard reporting format of meta-analyses from the Cochrane Collaboration network [19] . Continuous variables were evaluated using weighted mean difference (WMD), and binary variables were analyzed using the risk ratio (RR) and hazard ratio (HR). Median and range data were properly converted into means and standard deviations by adopting the method proposed by Hozo et al. [20]. The degree of heterogeneity, which indicated variance between studies, was assessed using the Higgins *I* ^2^ statistics and *Q* test [21]. The fixed-effect model was first fitted for all outcomes if the *p* value of the heterogeneity *Q* test was greater than 0.1 (*I* ^2^ ≤ 40); otherwise, the random effects model was used. Potential publication bias was assessed using Begg’s and Egger’s tests [22, 23]. Data analyses were performed using Review Manage version 5.1 (RevMan 5.1) software downloaded from the Cochrane Library. The GRADE profiler software (version 3.6) was used to estimate the level of evidence.

## Results

### Study selection and characteristics

**Fig. 1:**
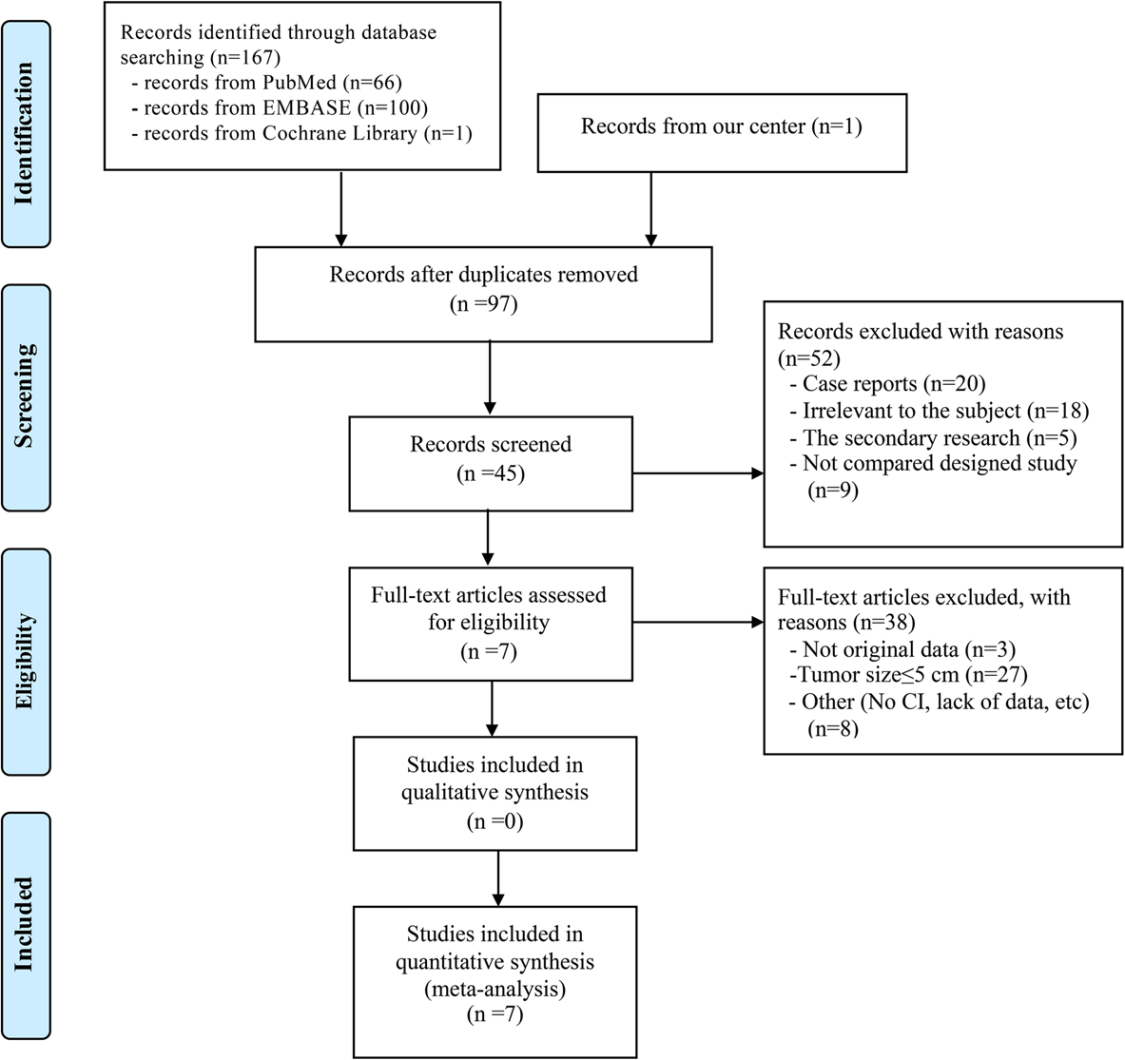
Flow chart of the literature search strategies

The primary search strategy retrieved 167 relevant English publications. After browsing titles and abstract, the duplicate records and the studies obviously did not meet the inclusion criteria were excluded, and 45 comparative studies remained. The remaining studies that did not conform to our research theme (tumor size >5 cm) and other criteria were excluded after we reviewed the full texts. In total, 6 accessible observational studies [7, 24–28] and one unpublished pair-matched retrospective cohort study from our center were included in the final analysis. A flow chart illustrates the detail search strategy (Fig. 1).

The basic feature and methodological quality evaluation points of the eligible studies are shown in Table 1. A total of 417 patients were enrolled in the meta-analysis, including 203 (48.7%) patients underwent laparoscopic resection and 214 (51.3%) patients underwent open resection. The MINORS evaluation criteria demonstrated that all of the original studies achieved high quality standards (points > 16). Table 2 summarizes the comparison results of baseline characteristics between the two groups. All of the baseline indicators were statistically comparable between the two groups (*P* > 0.05), and most of the baseline indicators were homogeneous, expect tumor size (*I* ^2^ = 69%). The relevant information on the use of neoadjuvant or adjuvant therapy is listed in Additional file 4. The available data of included studies did not show the significant difference in neoadjuvant or adjuvant therapy between the laparoscopic and open resection.

**Table 1:** Summary of studies included in the meta-analysis

Reference	Year of study	Country	Study design	group	sample size	Mean/median size (cm)	Median FU(range, mo)	Quality score
Kim [25]	2012 (1998–2011)	Korea	Retro	LAP	24	6.1	62.6(8.9–164.4)	17.5
				OPEN	14	7.2	58.3(18.8–123.2)	
Lin [17]	2014 (2007–2012)	China	Retro	LAP	23	7.2	34.0(6–78)	18
				OPEN	23	7.3		
Hsiao [26]	2014 (2002–2012)	Taiwan	Retro	LAP	18	6.3	37.2(16.8–133.2)	17
				OPEN	21	6	67.2(12.0–133.2)	
Takahashi [27]	2015 (1995–2011)	Japan	Retro	LAP	15	5.5	57(7–120)	16.5
				OPEN	12	7.5	69(13–154)	
Piessen [28]	2015 (2001–2013)	France	Retro	LAP	90	NA	NA	17.5
				OPEN	93			
Chun [29]	2016(2002–2015)	Singapore	Retro	LAP	23	6	20.5(0–163)	17
				OPEN	36	6	78(2–151)	
Our own study	2015(2008–2015)	China	Retro	LAP	13	6	48(26–78)	17.5
				OPEN	13	6	42(11–83)	

*Retro* retrospective observational study, *LAP* laparoscopic resection, *OPEN* open resection, *FU* follow up, *mo* months, *NA* not available

**Table 2:** Results of meta-analysis comparing baseline characteristics between LAP and OPEN

Baseline characteristic	Studies	LAP	OPEN	Heterogeneity	Overall	95% CI of	P
				(*P, I* ^2^)	effect size	overall effect	
Gender (male/female)	6	56/57	64/58	0.37, 0%	OR = 0.85	0.50, 1.43	0.53
Age	5			0.24, 27%	WMD = −2.29	−6.24, 1.65	0.25
Tumor size	6			<0.05, 69%	WMD = −0.54	−1.23, 0.15	0.13
Tumor location							
Upper /Middle	5	47/33	52/64	0.23, 29%	OR = 1.27	0.66, 2.42	0.47
Upper/ Lower	5	47/15	52/8	0.27, 23%	OR = 0.64	0.25, 1.66	0.36
Mitotic index (≤5/>5)	5	55/31	68/40	0.69, 0%	OR = 0.96	0.52, 1.75	0.89
Risk classification							
Intermediate/High	5	41/27	36/37	0.57, 0%	OR = 1.58	0.81, 3.12	0.18

*LAP* laparoscopic resection, *OPEN* open resection

### Intraoperative and postoperative outcomes

**Fig. 2:**
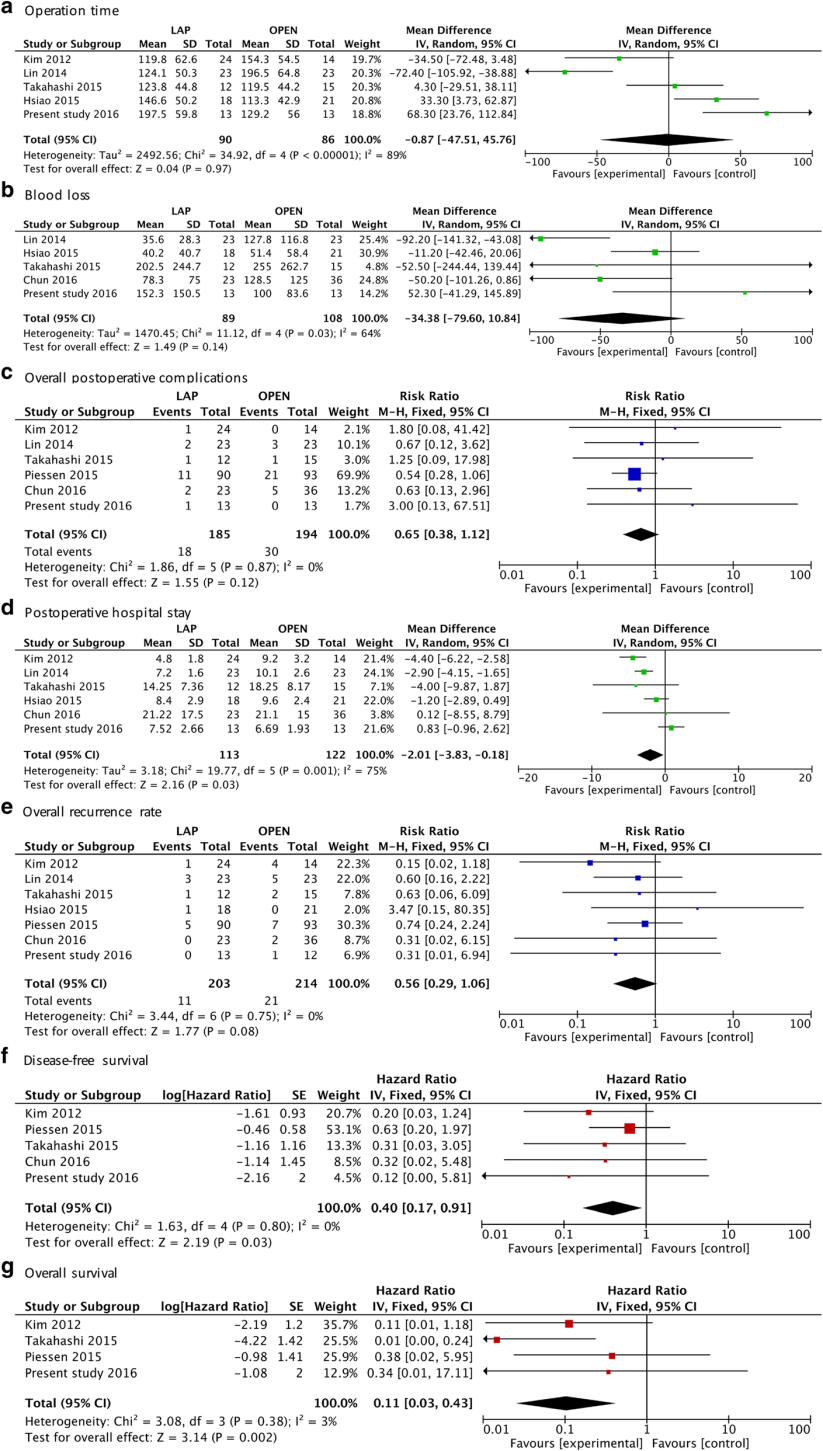
Forest plots illustrating the meta-analysis of the pooled data. (**a**) Operative time, (**b**) Intraoperative blood loss, (**c**) Overall postoperative complications, (**d**) Postoperative hospital stay, (**e**) Overall recurrence rate, (**f**) Disease-free survival, and (**g**) Overall survival

Six and 5 studies reported intraoperative blood loss and operative time, respectively. The present analysis revealed no significant difference in the operative time (WMD = −0.87 min; 95% CI: -47.50 to 47.75; *P* = 0.97) or blood loss (WMD = −34.38 ml; 95% CI: -79.60 to 10.84; *P* = 0.14) between laparoscopic and open resection groups. The overall complication rates in the two groups were 9.8% and 15.0%, respectively. The difference between the rate of overall complications was not statistically significant (RR = 0.65; 95% CI: 0.38 to 1.12; *P* = 0.12). The meta-analysis suggested that the open resection group exhibited shorter hospital stays compared with laparoscopic resection (WMD = −2.01 days; 95% CI: -3.83 to −0.18 *P* = 0.03) (Fig. 2).

### Long-term oncological outcomes

All of the studies reported that the rates of recurrence in laparoscopic and open resection groups were 5.4% and 9.8%, respectively. The results of the meta-analysis indicated no significant difference in recurrence between the two groups (RR = 0.56; 95% CI: 0.29 to 1.06; *P* = 0.08). Sufficient data on 5-year DFS were retrieved from 5 studies (*n* = 333). We calculated the pooled hazard ratio (HR) using a method of data conversion [29]. The pooled analysis revealed a better DFS for the laparoscopic resection group than that in the open resection group (HR = 0.40; 95% CI: 0.17 to 0.91; *P* = 0.03). Meta-analysis of 4 studies (*n* = 294) suggested that laparoscopic resection was associated with a better OS compared to open resection (HR = 0.11; 95% CI: 0.03 to 0.43; *P* = 0.002) (Fig. 2).

### Publication bias

### GRADE quality of evidence

There were 7 outcomes indicators in this study: operation time, blood loss, postoperative complications, postoperative hospital stay, overall recurrence rate, DFS and OS. Table 3 shows the level of GRADE system of evidence of each outcome indicator and the reasons for increases and downgrade of the level.

**Table 3:** GRADE profile evidence of the included studies

Outcome indicator	No. of studies	Quality assessment					No. of patients		Effect		Quality	Importance
		Risk of bias	Inconsistency	Indirectness	Imprecision	publication bias	LAP	OPEN	Relative (95% CI)	Absolute		
Operation time	5	no serious risk of bias	very serious^a^	no serious indirectness	serious^b^	None	90	86		WMD −0.87 (−47.50~47.75)	⊕⊝⊝⊝ very low	IMPORTANT
Blood loss	5	no serious risk of bias	serious	no serious indirectness^a^	serious^b^	None	89	108		WMD −34.38 (−79.60~10.84)	⊕⊝⊝⊝ very low	IMPORTANT
Postoperative complications	6	no serious risk of bias	no serious inconsistency	no serious indirectness	serious^a^	Maybe^d^	185	194	RR 0.65 (0.38~1.12)^c^		⊕ ⊕ ⊝⊝ low	CRITICAL
Postoperative hospital stay	6	no serious risk of bias	very serious^a^	no serious indirectness	serious^b^	None	113	122		WMD −2.01 (−3.83~ − 0.18)	⊕⊝⊝⊝ very low	IMPORTANT
Overall recurrence rate	7	no serious risk of bias	no serious inconsistency	no serious indirectness	serious^a^	None	203	214	RR 0.56 (0.29~1.06)^c^		⊕ ⊕ ⊕⊝ moderate	CRITICAL
Disease-free survival	5	no serious risk of bias	no serious inconsistency	no serious indirectness	no serious imprecision	None	162	171	HR 0.40 (0.17~0.91)		⊕ ⊕ ⊕⊝ moderate	CRITICAL
Overall survival	4	no serious risk of bias	no serious inconsistency	no serious indirectness	serious	None	149	158	HR 0.11 (0.03~0.43)		⊕ ⊕ ⊕⊝ moderate	CRITICAL

*LAP* laparoscopic resection, *OPEN* open resection

^a^have serious heterogeneity(*I* ^2^ > 75%)

^b^the sample size of included patients is too small

^c^the confidence interval of RR include 1

^d^exist publication bias

## Discussion

Surgical resection is the standard first-line therapy for gastric GISTs [14]. Advances in laparoscopic stapling devices and surgical technique [30] expanded laparoscopic resection as a minimally invasive surgery and an appealing alternate to open surgery with the potential advantage of requiring smaller incisions and less bowel manipulation. Several recent studies [31–33] reported the successful laparoscopic resection of tumors larger than 5 cm, including tumors up to 10 cm. However, the practice guidelines of ESMO clearly discourage a laparoscopic approach in patients with larger tumors because of the risk of tumor rupture [6]. Laparoscopic resection for gastric GISTs larger than 5 cm is also not recommended in the clinical practice guidelines for GISTs in Japan [15]. Therefore, the meta-analysis investigated the safety, feasibility, and long-term oncological outcomes of laparoscopic resection for gastric GIST size ≥5 cm. We found that laparoscopic resection was a safe and feasible approach for large-sized gastric GISTs regardless of technical or oncological aspects, and this approach achieved superior long-term oncological outcomes compared to open resection.

Under the premise of the merged comparable baseline characteristics, our review found no significant difference in blood loss, operation time and overall postoperative complications between open and laparoscopic resection, expect for a longer hospital stay in the laparoscopic resection group. The similar pooled outcomes of operation time and the postoperative complications had been repeatedly proven by some systematic reviews compared the two surgical approaches for gastric GISTs with the tumor size of all range (≤5 cm and >5 cm) [11, 13, 34]. Koh et al. [11] and Chen et al. [13] even indicated a reduced blood loss and lower incidence of complications in laparoscopic group. Our results further confirmed that laparoscopic resection does not increase the risk of the laparoscopic resection for gastric GISTs when the tumor size was >5 cm. The contradictory pooled outcome of postoperative hospital stays could be explained by the serious heterogeneity within the included studies, in view of the potential superiority of laparoscopic resection—smaller incisions and less bowel manipulation, could facilitate recovery and earlier discharge from the hospital.

The main concern of a laparoscopic approach for large scale tumors is the risk of tumor rupture, which causes a very high incidence of relapse [6]. Our review suggested that laparoscopy for gastric GISTs larger than 5 cm is a safe and feasible choice. The meta-analysis revealed a tendency for lower recurrence rates in laparoscopic resection patients (Fig. 2), but no significant difference was found between the two groups, which was consistent with the Ye et al.’ study [35], and they suggest a surgeon’s experience and skill must be considered prior to selecting the laparoscopic procedure to avoid rupture. The pooled long-term oncological outcomes in the present meta-analysis favored a laparoscopic approach with a better 5-year DFS and OS for gastric GISTs ≥5 cm. Since Koh et al. [11] had presented the comparable RFS and OS rates of two surgical approaches for gastric GISTs (tumor size range from 2.0–9.2), the results of the present meta-analysis could be a reference for a favorable prognosis of the laparoscopic approach for large gastric GISTs (≥5 cm).

To the best of our knowledge, the decision to proceed with a laparoscopic approach should be based on a variety of factors, including patient characteristics, tumor size, location, and the surgeon’s skills and experience [7]. All oncological principles of GIST resection must be followed to achieve the feasibility and safety of laparoscopic resection for gastric GISTs larger than 5 cm. The primary concern during laparoscopy is maintaining the integrity of the tumor. It is imperative to avoid grasping, and a portion of the dissected gastric wall and normal tissues around the tumor may be used as a handle for further dissection [25] to carefully move the tumor away from the jaws of the stapler and prevent tumor rupture. An endo-bag should be used routinely when removing tumors from the abdominal cavity.

This study has some inevitable limitations. The essential selection bias of the non-randomized and retrospective studies included in the meta-analysis may contribute to some incomparability between the two groups. A lower proportion of perioperative complications and postoperative recurrence was observed with laparoscopic resection, but the difference did not reach statistical significance. This result may be explained by type II error caused by the relatively small sample size of most enrolled studies. It is necessary to conduct randomized controlled trials or nonrandomized prospective studies of high quality to strengthen the evidence and confirm the status of laparoscopic resection for the larger gastric GISTs.

The GRADE Quality Assessment noted 4 outcome indicators of low or very low level evidence because of the following reasons: 1. serious heterogeneity between the studies was observed (*I* ^2^ > 75%), which leads to inconsistency in the meta-analysis; 2. the small sample size of included original studies and no statistical significance confidence interval of RR resulted in the imprecision of the study; and 3. the existence of publication bias of some outcomes. The indexes of postoperative complications, overall recurrence rate, disease-free survival and overall survival were “critical” outcomes, and the remaining outcome was “important”. The recommended grade was “weak” because of the relatively poor quality of the original research and the bias from observational research itself, which may impact the authenticity of the conclusion.

## Conclusion

Laparoscopic resection is a technically and oncologically safe and feasible approach for large-sized gastric GISTs compared to open resection. Laparoscopic resection should be a preferable choice based on the comprehensive meta-analysis, which demonstrated that laparoscopic resection achieved at least similar postoperative outcomes and superior oncological outcomes compared with those for open resection for gastric GIST larger than 5 cm in size.

## Additional files

Additional file 1: Search Strategy in Detail. (DOCX 14 kb)

Additional file 2: Results of Our Institution. (DOCX 18 kb)

Additional file 3: Minor Items. (DOCX 12 kb)

Additional file 4: Adjuvant or Neoadjuvant. (DOCX 36 kb)

## Abbreviations

CI: confidence interval
DFS: disease-free survival
GISTs: gastrointestinal stromal tumors
ICC: interstitial cells of Cajal
LAP: laparoscopic resection
NIH: National Institutes of Health
OPEN: open resection
OS: over-all survival
