# Supplementary material for: Meta-analysis comparing laparoscopic versus open resection for gastric gastrointestinal stromal tumors larger than 5 cm
Source: BMC Cancer. 2017 Nov 13;17:760. doi: 10.1186/s12885-017-3741-3 (PMC5683318; doi:10.1186/s12885-017-3741-3)
Supplement: Supplementary file 1 — Search Strategy in Detail. (DOCX 14 kb) [file 12885_2017_3741_MOESM1_ESM.docx]

Details of the search strategy:

Two authors (XL and MG) utilize the search strategies for each online database defined by (FF) to independently screened potentially eligible studies.

Pubmed

Performed:12/11/2016

gastric[title] AND (gastrointestinal stromal tumor[title] OR gastrointestinal stromal tumors[title] OR GISTs[title] OR GIST[title]) AND (open[title] OR laparoscopic[title] OR laparoscopy[title]) AND (resection[title] OR surgery[title])

N=66

EMBASE

Performed:12/11/2016

‘gastric’:ti AND (‘gastrointestinal stromal tumor’:ti OR ‘gastrointestinal stromal tumors’:ti OR ‘GISTs’:ti OR ‘GIST’:ti) AND (‘open’:ti OR ‘laparoscopic’:ti OR ‘laparoscopy’:ti) AND (‘resection’:ti OR ‘surgery’:ti)

N=100

Cochrane:

Performed:12/12/2016

gastric AND ("gastrointestinal stromal tumor" OR "gastrointestinal stromal tumors" OR "GISTs" OR "GIST") AND (open OR laparoscopic OR laparoscopy) AND (resection OR surgery)

N=1
