# Supplementary material for: Meta-analysis comparing laparoscopic versus open resection for gastric gastrointestinal stromal tumors larger than 5 cm
Source: BMC Cancer. 2017 Nov 13;17:760. doi: 10.1186/s12885-017-3741-3 (PMC5683318; doi:10.1186/s12885-017-3741-3)
Supplement: Supplementary file 2 — Results of Our Institution. (DOCX 18 kb) [file 12885_2017_3741_MOESM2_ESM.docx]

Table Demographic and clinicopathologic characteristics of present study

| Baseline characteristic | LAP(n=13) | OPEN(n=13) | *P* value |
| --- | --- | --- | --- |
| Gender(male/female) | 5/8 | 4/9 | 1.000 |
| Age(years) | 56.15±12.84 | 60.73±9.32 | 0.337 |
| Tumor size(cm) | 6(5-11) | 6(5-11) | 0.978 |
| Tumor location |  |  | 1.000 |
| Upper | 9(69.2%) | 9(69.2%) |  |
| Middle | 2(15.4%) | 3(23.1%) |  |
| Lower | 2(15.4%) | 1(7.7%) |  |
| Growth pattern |  |  | 1.000 |
| Intragastric | 11(84.6%) | 10(90.0%) |  |
| Extragastric | 2(15.4%) | 1(9.1%) |  |
| Mitotic index |  |  | 0.691 |
| ≤5 | 6(46.2%) | 5(38.5%) |  |
| >5 | 7(53.8%) | 8(61.5%) |  |
| Risk classification |  |  | 0.234 |
| Intermediate | 7(53.8%) | 4(30.8%) |  |
| High | 6(46.2%) | 9(69.2%) |  |
| CD117 |  |  | 1.000 |
| Positive | 0(0.0%) | 1(7.7%) |  |
| Negative | 13(100.0%) | 12(92.3%) |  |
| CD34 |  |  | 0.480 |
| Positive | 0(0.0%) | 2(15.4%) |  |
| Negative | 13(100.0%) | 11(84.6%) |  |
| Adjuvant imatinib treatment | |  | 0.234 |
| No | 7(53.8%) | 9(69.2%) |  |
| Post-operative | 6(46.2%) | 4(30.8%) |  |

LAP: laparoscopic resection, OPEN: open resection

Numerical variables are indicated as the means±SD or the median (range)

| Surgical outcome characteristics | LAP(n=13) | OPEN(n=13) | *P* value |
| --- | --- | --- | --- |
| Type of resection |  |  | 0.029 |
| Limited Resection | 4(30.8%) | 10(76.9%) |  |
| Proximal gastrectomy | 4(30.8%) | 3(23.1%) |  |
| Distal gastrectomy | 2(15.4%) | 0(0.0%) |  |
| Total gastrectomy | 3(23.1%) | 0(0.0%) |  |
| Operation time(min) | 197.46±59.774 | 129.23±.04556 | 0.006 |
| Blood loss(ml) | 100(30-600) | 50(20-300) | 0.315 |
| Time to first flatus(days) | 3.69±0.75 | 3.92±1.66 | 0.782 |
| Time to liquid diet(days） | 4.77±1.48 | 4.23±2.45 | 0.505 |
| Postoperative hospital stay(days) | 7.92±2.66 | 6.69±1.93 | 0.190 |
| Complications | 1(7.7%) | 0(0%) | 1.000 |
| Survival outcomes |  |  |  |
| Recurrence | 0 | 1 | 1.000 |
| Distant metastasis | 0 | 1 |  |

Table Surgical Procedure and Outcomes of Patients

LAP: laparoscopic resection, OPEN: open resection

Numerical variables are indicated as the means±SD or the median (range)

Statistical Analysis

Numerical variables are indicated as the means±SD or the median (range). Student’s t test or the Mann-Whitney *U* test was used for intergroup comparisons. Statistical analyses mentioned above were processed using SPSS, version 19.0 software (SPSS Inc, Chicago, United States). The gmatch macro of SAS 9.2 (SAS institute, Cary, NC, USA ) written by Erik Bergstralh & Jon Kosanke (http://www.mayo.edu/research/departments-divisions/department-health-sciences-research/division-biomedical-statistics-informatics/software/locally-written-sas-macros) was used to execute this pair matching process. P < 0.05 was considered statistically significant.
