# Supplementary material for: Meta-analysis comparing laparoscopic versus open resection for gastric gastrointestinal stromal tumors larger than 5 cm
Source: BMC Cancer. 2017 Nov 13;17:760. doi: 10.1186/s12885-017-3741-3 (PMC5683318; doi:10.1186/s12885-017-3741-3)
Supplement: Supplementary file 3 — Minor Items. (DOCX 12 kb) [file 12885_2017_3741_MOESM3_ESM.docx]

Appendix 3 the revised and validated version of MINORS

| Methodological items for non-randomized studies |  |
| --- | --- |
| 1.A clearly stated aim |  |
| 2.Inclusion of consecutive patients |  |
| 3.Prospective collection of data |  |
| 4.Endpoints appropriate to the aim of the study |  |
| 5.Unbiased assessment of the study endpoint |  |
| 6.Follow-up period appropriate to the aim of the study |  |
| 7.Loss to follow up less than 5% |  |
| 8.Prospecrive calculation of the study size |  |
| *Additional criteria in the case of comparative studies* |  |
| 9. An adequate control group |  |
| 10.Contemporary groups |  |
| 11. Baseline equivalence of groups |  |
| 12. Adequate statistical analyses |  |
