# Supplementary material for: Meta-analysis comparing laparoscopic versus open resection for gastric gastrointestinal stromal tumors larger than 5 cm
Source: BMC Cancer. 2017 Nov 13;17:760. doi: 10.1186/s12885-017-3741-3 (PMC5683318; doi:10.1186/s12885-017-3741-3)
Supplement: Supplementary file 4 — Adjuvant or Neoadjuvant. (DOCX 36 kb) [file 12885_2017_3741_MOESM4_ESM.docx]

|  | LAP | OPEN | P value |
| --- | --- | --- | --- |
| Kim[25] | NA | |  |
| Lin[17] |  |  | 0.811 |
| No | 8 | 6 |  |
| Post-operative | 14 | 16 |  |
| Pre- and post-operative | 1 | 1 |  |
| Hsiao[26] | NA | |  |
| Takahashi[27] | |  | 0.294 |
| No | 14 | 9 |  |
| Post-operative | 1 | 3 |  |
| Piessen[28] | NA | |  |
| Chun[29] | NA | |  |
| Our own study | |  | 0.428 |
| No | 6 | 9 |  |
| Post-operative | 6 | 4 |  |

Table the situation of neoadjuvant or adjuvant therapy of the included studies.
